# Impacts of artificial intelligence on computed tomography in endodontics: an integrative review

**DOI:** 10.1186/s12903-026-07967-7

**Published:** 2026-02-28

**Authors:** Camila da Silva Fagundes, Ana Ligya Monteiro Soares, Fábio Luiz Cunha D’Assunção, Elsbeth Kalenderian, Cláudia Batista Mélo

**Affiliations:** 1 Undergraduate Degree in Dentistry, Federal University of Paraíba, Paraiba, Brazil; 2 Department of Restorative Dentistry, Federal University of Paraíba, Paraíba, Brazil; 3 Department of Surgical and Diagnostic Sciences, Marquette University School of Dentistry, Milwaukee, USA; 4 Department of Clinical and Social Dentistry, Federal University of Paraíba, Paraiba, Brazil; 5 Federal University of Paraíba, Conjunto Presidente Castelo Branco III, s.n., Castelo Branco, João Pessoa, Paraíba, 58050-585 Brasil

**Keywords:** Artificial intelligence, Computed tomography, Endodontics, Clinical relevance, Therapeutic planning

## Abstract

**Background:**

Artificial Intelligence (AI), defined as systems capable of replicating human tasks aimed at performing complex tasks and supporting decision-making, is entering the field of Dentistry, standing out with its application in research and clinical practices, such as diagnosis using Cone Beam Computed Tomography. The objective of this study is to identify and organize scientific evidence related to the use of Artificial Intelligence in computed tomography as an advanced resource in Endodontics.

**Materials and methods:**

This study is an integrative review, structured in well-defined steps, namely: formulation of the guiding question, selection of inclusion and exclusion criteria, identification of descriptors for creating the search strategy in English and Portuguese, and selection of databases Scopus, Web of Science, PubMed, LILACS, and Google Scholar. Articles addressing the application of Artificial Intelligence in Computed Tomography in Endodontics were included, without distinction of ethnicity and/or sex of patients. Duplicates and content unrelated to the core topic were excluded.

**Results:**

A total of 20 articles were included for full analysis and reading. The analysis highlights the variety of benefits, objectives, artificial intelligence models, individualities in tomography categories used, and a plurality of study limitations presented in the articles.

**Conclusion:**

The application of Artificial Intelligence (Al) in the analysis of tomographic images in Endodontics show great potential for use for patient diagnosis and therapeutic planning.

**Supplementary Information:**

The online version contains supplementary material available at 10.1186/s12903-026-07967-7.

## Introduction

Artificial Intelligence (Al) technologies are integrated into various domains of society, such as economics, politics, and healthcare [1]. Similar to other healthcare professions, Dentistry is undergoing a transitional period of incorporating and adapting to emerging technologies that may enhance practices and processes in the sector. AI, defined by systems capable of replicating human tasks to perform complex actions and support decision-making, has entered Dentistry, particularly in research and clinical applications [1–3].

The relevance of Al in Dentistry can be explained through understanding how these systems operate. There are different types and subdivisions of Al systems that all function by involving three core abilities: learning, reasoning, and self-correction [4]. The complexity with which a system operates these three abilities classifies it as either Machine Learning (ML) or Deep Learning (DL) which is a subset of machine learning [4]. ML represents a computational learning method that relies on human intervention for data input and information processing, while DL operates with more advanced processes, capable of autonomously learning representations without human intervention, making it more effective in data processing [5]. In short, machines learn by being exposed to datasets, determining patterns, and enabling effective solutions and predictions [2].

Given this, the potential of Al-driven systems offers important benefits to Dentistry, such as simplifying care and improving the quality of patient services [1, 6]. Rapid and accurate analysis of large volumes of data for diagnosis and treatment contributes to transforming the profession into a more personalized, preventive, and participatory practice [3, 6, 7]. Precise diagnosis, which combines proper clinical examination with complementary imaging tests such as radiographs and computed tomography, is fundamental in Dentistry [1, 8, 9].

In Endodontics, image processing is the basis for planning and executing treatments in all cases [10]. In clinical practice, dentists commonly deal with patients presenting with various symptoms, often requiring critical image assessments for diagnostic confirmation [11]. AI-based systems have shown great potential when used to detect prevalent dental conditions such as caries, periapical lesions, and periodontal bone loss [11]. Therefore, the use of AI is bound to enhance our ability to decide on the correct procedure, agility in the clinical decision-making process, and predictability of therapeutic success for dentists [3, 7, 12, 13].

Al algorithms in computed tomography image analysis have enabled significant advances, including diagnostic accuracy in obtaining information on root canal system anatomy, measuring working length, analyzing and detecting periapical lesions and root fractures, predicting postoperative pain, and estimating success rates in retreatment case [3, 7, 11, 12, 14].

Studies highlight 3D photorealistic reconstructions of root canal space and dental anatomy, a technique known as Cinematic Rendering (CR) [15]. Additionally, Russian companies have already developed software to assist professionals in analyzing CT scans to determine root canal morphology correctly [16].

Other studies demonstrate that DL algorithms can accurately evaluate root canal curvatures, define their three-dimensional changes after instrumentation, locate the minor apical constriction [16] and facilitate image analysis to identify landmarks in two-dimensional (2D) or three-dimensional (3D) examinations [4]. Additionally, ML processing techniques have shown high-performance results in classifying dental caries in radiographs [17] and automating non-invasive differential diagnoses of periapical lesions, such as dental granulomas and radicular cysts in computed tomography scans [13, 17–19].

Despite the incorporation of AI technologies driving the advancement of Dentistry through imaging technology improvements and contributing directly to Endodontics evolution, few studies provide systematic scientific evidence on how Al-based technologies are applied in daily clinical practice and their role in reducing diagnostic errors in Endodontic CT scans. Therefore, understanding the factors that still limit the integration of Al into dentists’ daily clinical practice is crucial.

Given this, the objective of this study is to identify and organize scientific evidence related to the use of Artificial Intelligence in computed tomography as innovative resource in Endodontics.

## Materials and methods

### Study design

This study consists of an integrative review conducted following the reporting items of the PRISMA 2020 (Preferred Reporting Items for Systematic Reviews and Meta-Analyses) guide, ensuring a reproducible, rigorous, and transparent method for mapping evidence from scientific literature and gray literature, based on a current scientific question that contributes to the advancement of knowledge. It encompasses the absence of protocol registration in international databases – PROSPERO – and followed the systematic steps of identification, selection, extraction, and synthesis of evidence.

### Identification and research question

The guiding question was formulated in a clear and specific manner, according to the PICO strategy (Table 1).

**Table 1:** The pico strategy. Table illustrating the PICO strategy used in the study, 2025

Component	Description
P (Population)	Cone beam computed tomography (CBCT) images with endodontic interest.
I (Intervention)	Use of systems based on Artificial Intelligence (AI), including machine learning (ML) and deep learning (DP)
C (Comparison/Control)	Not applicable
O (Outcome)	Applications, diagnostic or operational performance in endodontics

Based on this guideline, the question was defined as follows: “What are the applications of Artificial Intelligence in the analysis of computed tomography scans in endodontics?”

### Eligibility criteria

The inclusion and exclusion criteria were established based on the guiding question. Original in vitro research studies addressing the application of Artificial Intelligence in Computed Tomography in Endodontics were included in this review, without distinction of ethnicity and/or gender of patients. Evidence from national and international literature was included, with no specific restrictions on language or date of publication. Duplicates, conference abstracts, theses, literature reviews, and articles not related to the three pillars of the core theme—endodontics, computed tomography, and artificial intelligence—were excluded.

### Sources of information or databases used

The selection of scientific publications took place in February 2025. The searches were conducted in the following databases: Scopus, Web of Science, PubMed, and Latin American and Caribbean Health Sciences Literature (LILACS), in addition to Google Scholar as a source of gray literature, using the first 100 prevalent articles. Restrictions were imposed for the Portuguese and English languages, with no specific restrictions on publication date. Portuguese terms were used only for the LILACS and Google Scholar databases, together with the English strategy.

### Search strategy

The search strategy was constructed based on a search of previously defined keywords, using descriptors in Portuguese and English, according to the Health Sciences Descriptors (DeCS) and Medical Subject Headings (MeSH). Synonyms and related terms were applied to optimize the capture of relevant articles. The main concepts were combined with the Boolean operator AND. Synonyms within each concept were combined with the operator OR (Table 2). Searches were then performed in the following databases: Scopus, Web of Science, PubMed, and Latin American and Caribbean Health Sciences Literature (LILACS), in addition to Google Scholar as a source of gray literature, in which the first 100 results presented with the application of the search strategy were considered for the selection process.

**Table 2:** Search strategy. Illustrative table of the research process in the databases used in the study, 2025

Database	Selection period	Results
Google Scholar (gray literature)	15/02/2025	100
PubMed	15/02/2025	47
Scopus	15/02/2025	24
Web of Science	15/02/2025	21
Literatura Latino-Americana e do Caribe em Ciências da Saúde (LILACS)	15/02/2025	0
Search strategy in English	((“Endodontology” OR “Endodontics”) AND (“Tomographies” OR “Tomography”) AND (“Artificial Intelligence” OR “Acquisition, Knowledge (Computer)” OR “Computational Intelligence” OR “Computer Reasoning” OR “Computer Vision System” OR “Computer Vision Systems” OR “Intelligence, Artificial” OR “Intelligence, Computational” OR “Intelligence, Machine” OR “Knowledge Acquisition (Computer)” OR “Knowledge Representation (Computer)” OR “Knowledge Representations (Computer)” OR “Machine Intelligence” OR “Reasoning, Computer” OR “Representation, Knowledge (Computer)” OR “System, Computer Vision” OR “Systems, Computer Vision” OR “Vision System, Computer” OR “Vision Systems, Computer”))	
Search strategy in Portuguese	((“Endodontia” OU “Endodontologia”) E (“Tomografia”) E (“Inteligência Artificial” OU “Aquisição de Conhecimento (Computador)” OU “Computational Intelligence” OU “Raciocínio Automático” OU “Raciocínio Computacional” OU “Representação de Conhecimento (Computador)” OU “Representação do Conhecimento (Computador)” OU “Sistemas de Visão Artificial” OU “Sistemas de Visão Computacional”))	

### Selection process

The identified studies were organized in the EndNote Web reference manager (Clarivate Analytics, USA), which was used to remove duplicates. The selection process was conducted independently and blindly by two reviewers (double-blind). The articles were imported into Rayyan software (rayyan.ai), an artificial intelligence-based web tool developed by the Qatar Computing Research Institute (QCRI), Qatar. Initial screening was performed by reading titles and abstracts. The full texts of studies considered potentially eligible were retrieved and reevaluated for inclusion criteria. All disagreements between reviewers were resolved through discussion or, when necessary, arbitration by a third reviewer to provide a final opinion. The complete flow of the process is presented in the PRISMA 2020 Diagram.

### Data collection process

The data from the included studies were extracted independently by the same two reviewers and organized into standardized tables and flowcharts developed in Microsoft Excel to facilitate understanding and analysis of the findings. Disagreements in extraction were resolved by consensus. As this was an integrative review based on public domain literature, approval by the Research Ethics Committee was not required. It was not necessary to contact the authors of the primary studies to obtain additional data. The authors declared no conflicts of interest or external funding for this study.

### Data items

For each included study, information was extracted on the author, year and country of publication, type of imaging exam, and purpose of the application of artificial intelligence. In addition, the type of task performed by the model, AI architecture used, reported benefits for endodontics, and methodological limitations described by the authors were also extracted.

### Assessment of study bias risk

In this review, we chose not to perform a formal assessment of bias risk using standardized scales due to the extreme methodological heterogeneity and predominantly pilot nature of the included studies. The current literature on AI applied to Cone Beam Computed Tomography (CBCT) in Endodontics reveals a significant disparity in model architecture, variability in sample size, and often the absence of independent external validation. This methodological fragmentation prevents a fair quantitative comparative synthesis, since the ‘ground truth’ criteria and performance metrics vary substantially between experiments, characterizing the field as still in an exploratory stage.

## Results

**Fig. 1:**
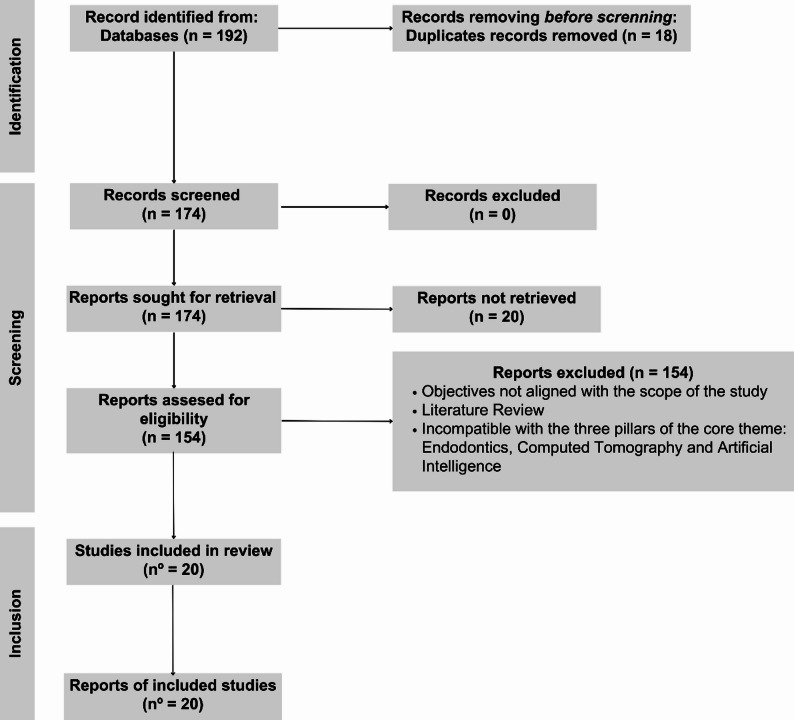
Illustrative flowchart of the selection process of articles included in the study, 2025.

Based on searches in established databases, a total of 192 publications were identified, of which 18 duplicates were eliminated, leaving 174 publications for title and abstract analysis. Through reading titles and abstracts, 154 papers were excluded, leaving 20 papers for full-text reading. The remaining 20 articles were evaluated for eligibility and corresponded to the guidelines of the base theme and the inclusion criteria. No exclusions were made after reading the 20 articles in full. The study selection process is summarized in Fig. 1, with specific information such as author, year, country, benefits for endodontics, AI application objectives, type of AI used, types of tomography used, and study limitations summarized in a supplementary file.

### General characteristics of the studies

The studies were published between 2020 and 2024, with a diverse geographical distribution, including North America, South America, Europe, and Asia. All used Cone Beam Computed Tomography (CBCT) as the image acquisition method, applied to the endodontic context. Most studies had an observational design, with retrospective analysis of image databases.

### Objectives of the application of artificial intelligence in cone beam computed tomography

The application of Artificial Intelligence focused mainly on tasks of detection, classification, and segmentation of endodontic structures and conditions. Among the main objectives described are the identification of periapical lesions, the detection of endodontic technical errors, the automatic segmentation of root canals, and their classification as healthy or pathological.

### Artificial intelligence models used

The most frequently used AI architectures were Convolutional Neural Network (CNN), including U-Net and Siamese network models, as well as hybrid systems combining different deep learning approaches. Some studies do not specify the architecture used in detail, while others have proposed customized models for application in CBCT images.

### Performance and reported benefits of using AI in endodontics

In general, studies reported that AI models performed satisfactorily for the proposed tasks, with the potential to assist in endodontic diagnosis, increase accuracy in identifying anatomical structures, and reduce interobserver variability. The benefits described included support for clinical decision-making and greater consistency in image interpretation.

### Performance metrics

The included studies reported high performance metrics for various tasks. For detection and classification, the models achieved sensitivity between 72.2% [20] and 100% [21] and specificity between 66.7% [21] and 100% [22]. In the segmentation of anatomical structures, Dice scores (DSC) ranged from 0.67 [10] for lesions to 0.99 [23] for the image background, with typical values above 0.85 [24, 25] for teeth and root canals.

### Methodological limitations

The main limitations reported involved restricted databases, the presence of artifacts in CT images, limitations in the number of cases analyzed, and the absence of external validation in some studies.

## Discussion

This review of 20 studies on Artificial Intelligence in Cone Beam Computed Tomography for Endodontics highlights that accurately detecting periapical lesions in tomographic images requires both professional experience and time. Furthermore, the segmentation of anatomical structures, such as the pulp cavity and root canals is not trivial, especially for less experienced dentists. In this context, studies conducted in various countries highlight the variety of benefits for Endodontics in addressing these issues (see Additional file 1).

### Application of AI in endodontics

As evidenced in the summary of the included studies, studies conducted in China [20, 26–29], Canada [30] and the United Kingdom [25, 31], revealed that the application of an AI model in tooth and pulp cavity segmentation, as well as in the detection of periapical lesions, has the potential to increase diagnostic accuracy, reduce errors, and assist in appropriate therapeutic planning by the dentist, resulting in the main benefits observed in the reviewed articles. Other studies from the United States [22], presented as a major innovation the possibility of detecting and segmenting MB2 canals in endodontically treated maxillary molars through computed tomography images [22].

The studies underlined the importance of applying AI in computed tomography in alignment with expected benefits aiming at the evaluation, validation, and optimization of the tested Al model, mostly to ensure automated precision and standardization in the classification of periapical lesions. In this context, studies conducted in Belgium [24, 32], Austria [33], Poland [21], Brazil [34], the United Kingdom [25], China [28], and the United States [35], have also sought the development of proprietary tools to minimize manual interventions in clinical use, promoting innovations in the detection and segmentation of root canals, as exemplified in cases of C-shaped canals.

### Type of tomography

Regarding the types of tomography, Cone Beam Computed Tomography (CBCT) was predominantly used in almost all reviewed articles. A study from China [26] stands out for its use of Micro-CT, aimed at exponentially increasing the precision in the segmentation processes of teeth and the pulp cavity in an experimental setting. Compared to CBCT, Micro-CT presents even higher resolution, which contributed to improving neural network training for their designated tasks.

### Use of neural networks

This review mainly reports on the performance of the algorithm, and not necessarily on confirmed clinical results. However, this review demonstrates that the use of Convolutional Neural Networks (CNNs) in Endodontics has led to notable improvements in diagnosis and treatment planning. These networks, widely used in automated pattern detection, have been employed for the classification of dental structures, such as root canals and dental roots, based on images obtained from cone beam computed tomography [24–27, 31, 32, 34, 36, 37]. This advance signals greater standardization of endodontic planning, reducing errors and aiding in clinical decision-making.

In conjunction with CNNs, different variations of the U-Net architecture have been employed to optimize the segmentation of dental structures. Among these variations, PAL-Net (Phase Alternating Line) [36], Residual U-Net [35], Xception U-Net [35], Bayesian U-Net [38], and Swin-UNETR (Swin UNEt Transformers) [23] stand out. The use of these architectures contributes to the precise segmentation of dental structures, allowing detailed identification of the tissues constituting dental elements and promoting relevant advances in planning endodontic interventions, especially in cases of complex root anatomy.

Beyond CNN applications, analyzed studies demonstrated the use of deep learning techniques, such as Deep Learning and Transfer Learning, which allow the adjustment of pre-trained neural networks for the detection of specific patterns in dental images. Among the algorithms used, EfficientNet B7 [24, 37], DenseNet, VGG [34, 37], Inception [37], MobileNet [37], and ResNet [37] stand out. The study by Calazans et al. (2024) was the most comprehensive in exploring these techniques, demonstrating their effectiveness in standardizing and optimizing the evaluation of endodontic treatment assisted by pre-trained CNNs.

Other strategies were adopted to enhance the performance of AI models in Endodontics. Siamese Networks were used for tomographic image comparison [37], Monte Carlo Dropout and Bayesian U-Net were employed for uncertainty quantification associated with sample variations [38], Feature Pyramid Networks (FPN) improved the detection of dental structures at different scales [24], and Active Learning (AL) was applied to reduce the need for large volumes of labeled data in Al training, optimizing its performance with fewer samples [38].

Among the reviewed studies, the research by Zhao et al. (2024) stands out, developing PAINet, a proprietary deep learning-based system aimed at assessing apical periodontitis. This model enables more accurate diagnosis of periapical lesions, aiding in clinical decision-making, optimizing treatment planning, and suggesting appropriate therapeutic approaches for each analyzed endodontic case. However, it should be reiterated that most of the studies cited in this discussion are retrospective, in vitro, or conducted under controlled experimental conditions, not yet validated externally or by prospective clinical studies.

### Accuracy of AI use as a support tool in CBCT image screening

Furthermore, the high sensitivities and DSC values observed indicate that AI models are competent in identifying patterns and delimiting structures in CBCT images, demonstrating potential to serve as screening and image analysis support tools. However, the significant variation in metrics such as specificity and inferior performance in more complex tasks (e.g., DSC for lesions = 0.52) [10] reveal that diagnostic accuracy and robustness may be compromised in challenging scenarios, such as in the presence of artifacts or complex anatomies, limiting their direct and widespread application in current clinical practice.

### Limitations of studies on the application of AI in endodontics

The main limitations of studies applying AI in Endodontics include reduced sample sizes, the limited number of analyzed teeth, and the restriction to a single dental group. This approach hinders the generalization of developed systems, especially when applied to teeth with significantly different anatomies from those studied [21, 22, 24, 26–29, 31–35, 38]. The limitation of sample diversity and anatomical variation compromises the clinical applicability of these systems, as they were trained in restricted scenarios that do not fully reflect the complexity of Endodontics in daily practice.

Another relevant factor pointed out in some studies is the presence of image artifacts, such as metallic restorations, implants, and previous endodontic treatments, as well as image quality, distortions, and noise, which affect the accuracy of AI models. These limitations negatively impact the performance of diagnostic and endodontic planning tools [25, 28, 31, 32, 36, 37]. Thus, implementing these technologies in real clinical settings remains challenging, as their effectiveness directly depends on the quality of the generated tomographic image. Furthermore, the need to exclude patients with restorations or previous dental treatments that generate radiographic artifacts represents an additional obstacle to the widespread adoption of these tools in routine endodontics.

In view of this, the application of AI in Endodontics remains restricted to studies conducted with isolated teeth of similar anatomy, with high-quality images, and without the presence of artifacts. These limitations hinder the translation of these tools into daily clinical practice, as real cases involve teeth with varied anatomies and often present artifacts in tomographic images.

This research also has some limitations, such as assessing the heterogeneity of various studies, limited availability of certain AI tools, and the fact that it is an integrative review. However, it opens the door for future research covering prospective, multicenter, and larger data sets; external validation, open data sets, as well as clinical needs (workflow integration, interpretability, regulation, training for technicians, dentists, and clinics).

This research was limited to articles published in Portuguese and English. Despite the recognized scientific production in Endodontics in Spanish-speaking countries, the restriction aimed to maintain the interpretive rigor of the AI systems discussed. Future research with a multilingual scope is encouraged to validate the applicability of these models in different sociocultural and clinical contexts in Ibero-America.

Thus, although the results presented are promising, these methodological limitations and the absence of external validation in some of the studies make it difficult to transpose these tools into daily clinical practice, since real cases involve teeth with varied anatomies and often present artifacts in tomographic images. These studies reinforce the need for future research with more robust details, larger samples, and greater clinical representativeness in order to consolidate the use of AI as an effective support tool in endodontics.

## Conclusions

Respecting the limitations of this study, it was concluded that studies associating the use of artificial intelligence in the analysis of tomographic images for endodontic purposes show high potential for use, but there is a need for external validation and future prospective clinical studies to consolidate the use of this technology.

## Supplementary Information

Supplementary Material 1.

## Data Availability

All data generated or analysed during this study are included in this published article.
