# Supplementary material for: Impacts of artificial intelligence on computed tomography in endodontics: an integrative review
Source: BMC Oral Health. 2026 Feb 28;26:599. doi: 10.1186/s12903-026-07967-7 (PMC13059364; doi:10.1186/s12903-026-07967-7)
Supplement: Supplementary file 1 — Supplementary Material 1. [file 12903_2026_7967_MOESM1_ESM.pdf]

Author/Year/Country	Title	Benefit for Endodontics	Purpose of applying AI in CTFC	Type of Tomography	IA	Study limitations
Albitar et al., 2022. Estados Unidos.	"Artificial Intelligence (AI) for Detection and Localization of Unobturated Second Mesial Buccal (MB2) Canals in Cone-Beam Computed Tomography (CBCT)."	AI with the potential to identify obstructed MV2 canals in endodontically treated teeth.	Automatically detect and segment MV2 canals in endodontically treated maxillary molars in CBCT.	Cone Beam Computed Tomography	U- Net	Does not fully represent U-Net performance due to metric limitations; Inadequate and insufficiently heterogeneous sample size; Manual segmentation lacking sufficient precision; Limitations of the ITK-SNAP program (inability to correct CTFC planes).
Brignardello, 2020. Canadá.	"Artificial intelligence system seems to be able to detect a high proportion of periapical lesions in cone-beam computed tomographic images."	The AI system performs well in detecting periapical lesions.	Evaluate the accuracy of using an AI system to detect periapical pathology in cone beam computed tomography (CBCT) images.	Cone Beam Computed Tomography	He did not specifically mention it.	One of the flaws in the system was incorrect tooth numbering; authors did not describe a variety of clinical scenarios; researchers had only one radiologist diagnosing periapical lesions; researchers did not provide a description of the type of lesions included and did not state in the results whether the AI system diagnosed lesions where there were none.
Calazans et al., 2024. Brasil.	"A system for automatic classification of endodontic treatment quality in CBCT"	CNNs have the potential to be used as a support and standardization tool in assessing the quality of endodontic treatment in clinical practice.	Identify endodontic technical errors using Cone Beam Computed Tomography (CBCT) and compare the performance of the automatic classification system with that of dentists	Cone Beam Computed Tomography	Combination of AI Systems Concatenated Siamese Network, Convolutional Neural Networks (CNN),<br>Transfer Learning Techniques (Keras Package: DenseNet, EfficientNet, Inception, MobileNet, ResNet, VGG, Xception), Selected Networks (EfficientNet B1 and EfficientNet B7).	Dental materials, especially those used for endodontic filling, may have influenced the results. It was not possible to include clinical signs and symptoms and effects on the environment such as CBCT scans from an image database, and this information was not available.<br>Dentists evaluated the best sagittal and coronal reconstructions whose periapical status and endodontic treatment quality were visible, chosen by an oral and maxillofacial radiologist
Calazans et al., 2022. Brasil.	"Automatic classification system for periapical lesions in cone-beam computed tomography"	Introduction of an automatic classification system for dental diagnosis in cone beam computed tomography, in which coronal and sagittal sections are used for the detection of periapical lesions.	Classify teeth as healthy or with endodontic lesions using an automatic classification system based on Artificial Intelligence.	Cone Beam Computed Tomography	Siamese network combined with the use of convolutional neural networks with transfer learning for VGG-16 and DenseNet-121 networks	The lesions present in the images in the UFPE database are considered minor in the field of dentistry (although, in the study, a distinction was made between major and minor lesions for the purposes of analyzing the results), which makes classification even more complex. The results seem to point to a difficulty that arises from the distinction between teeth without lesions and those with minor lesions.
Chau et al., 2024. China.	"A novel AI model for detecting periapical lesion on CBCT: CBCT-SAM"	Provide assistance, through increased accuracy and diagnosis, and reduction of errors, at the specialist level in the identification of periapical lesions in CBCT.	Investigate the effectiveness of SBCCT-SAM, a new artificial intelligence (AI) model, in identifying periapical lesions in CBCT.	Cone Beam Computed Tomography	"CBCT-SAM, CBCT-SAM without Prediction Refinement Module (PPR), and two previously developed models: Modified U-Net and PAL-Net"	Limited data available; Small, non-standardized dataset; Non-standardized data may introduce noise, hindering the correct extraction of features and resulting in inaccurate tests; Limited intra-rater and inter-rater visibility may have led to errors in the gold standard data.
Chen et al., 2024. Estados Unidos.	"Leveraging Pretrained Transformers for Efficient Segmentation and Lesion Detection in Cone-Beam Computed Tomography Scans"	AI with excellent segmentation and detection of periapical lesions that provides an alternative with smaller training datasets compared to U-Net."	Evaluate the use of pre-trained transformers for segmentation of CBCT volumes in the detection of periapical lesions.	Cone Beam Computed Tomography	U-Net and Swin-UNETR (Swin-UNETR-PRETRAIN and Swin-UNETR-SCRATCH)	Low reliability in CT interpretation; Limited data set that may impair the model's ability to detect lesions; The study did not evaluate the worst-case model; Generalization of results by using only one CTFC model.
W.T. Fu et al., 2024. China.	"Clinically Oriented CBCT Periapical Lesion Evaluation via 3D CNN Algorithm"	Automatic, effective, and highly accurate detection of periapical lesions associated with apical periodontitis.	Automated detection and segmentation of PALs (periapical lesions) associated with AP (apical periodontitis) from CBCT images.	Cone Beam Computed Tomography	PAL-Net (new convolutional neural network algorithm - proposed deep 3D U-Net)	Variations in imaging protocols influenced image quality and resolution; challenges in capturing all relevant information in a dataset; the present datasets exclude any potential diseases other than PA-associated injuries (specific training)
Jiayu Huang MS et al., 2024. Estados Unidos.	Uncertainty-based Active Learning by Bayesian U-Net for Multi-label Cone-beam CT Segmentation	Greater segmentation and accuracy in detecting lesions in CT scans and reduced need for extensive labeling in AI training for images.	Create Active Learning (AL) strategies in AI training for segmentation and detection of periapical lesions in CBCTs, resorptions, or iatrogenic errors.	Cone Beam Computed Tomography	Bayesian U-Net, Monte Carlo Dropout (MC Dropout), and Active Learning (AL) algorithms	False negative detection results; Small sample size; and The results reflect only the improvements of the AL algorithms, not the full capabilities of the AI platform in dentistry.
Wojciech Kazimierczak et al., 2024. Polônia.	Endodontic Treatment Outcomes in Cone Beam Computed Tomography Images—Assessment of the Diagnostic Accuracy of AI	High diagnostic accuracy of AI platforms in evaluating endodontic treatment outcomes using CBCT images	Evaluate the diagnostic accuracy of the AI-driven Dignocat platform for assessing endodontic treatment outcomes using cone beam computed tomography (CBCT) images.	Cone Beam Computed Tomography	Diagnocat	Small sample size; Evaluation confined to an AI platform; Biased reference standard; Low detection for areas of voids and short fills; Retrospective study.

Kirnbauer et al., 2022. Áustria.	Automatic detection of periapical osteolytic lesions on cone-beam computed tomography using deep convolutional neuronal networks	Automated detection and segmentation of periapical lesions in CBCT	Develop and validate a fully automated, optimized deep CNN for the automated detection and segmentation of PALs in routine three-dimensional (3D) CBCT datasets.	Cone Beam Computed Tomography	Deep Convolutional Neural Networks – CNNs, SpatialConfiguration-Net (SCN), Modified U-Net	The limitations of the study include the limited number of cases, class imbalance, dependence on manual segmentation, exclusion of immature teeth, and influence of image quality.
Lahoud et al., 2021. Bélgica.	Artificial intelligence for fast and accurate 3-dimensional tooth segmentation on cone- beam computed tomography	Fast and accurate tooth segmentation in CBCT	Develop and validate a clinically functional AI-based dental segmentation tool capable of minimizing manual interventions and providing fast, accurate, and consistent results essential for clinical use.	Cone Beam Computed Tomography	Convolutional neural networks (CNNs), specifically a Feature Pyramid Network (FPN) with an EfficientNet-B7 encoder.	The limitations of the study include the lack of molar segmentation, the need to manually select the region of interest, and the segmentation of only one tooth at a time, requiring future optimizations.
Lin et al., 2022, China.	[Segmentation and accuracy validation of mandibular molar and pulp cavity on cone- beam CT images by U-net neural network]	The application of the U-Net model in pulp cavity segmentation in CBCT images improves diagnostic accuracy and assists in endodontic planning, reducing errors and analysis time.	Evaluating the accuracy of mandibular molar pulp cavity segmentation in cone beam computed tomography (CBCT) images using the U-Net neural network	Cone Beam Computed Tomography	Deep convolutional neural networks (CNNs) and U-Net architecture	The small sample size, anatomical variability of mandibular molars, and possible inaccuracies in segmentation due to CBCT image quality.
Lin et al., 2021. China.	Micro-Computed Tomography-Guided Artificial Intelligence for Pulp Cavity and Tooth Segmentation on Cone-beam Computed Tomography	The application improves the accuracy and automation of tooth and pulp cavity segmentation in CBCT, optimizing endodontic diagnosis and planning.	This study aims to use a new data stream based on microcomputed tomography (micro-CT) images to train the U-Net convolutional neural network for accurate segmentation of the pulp cavity and teeth in CBCT images.	Microcomputed Tomography (Micro-CT) and Cone Beam Computed Tomography (CBCT)	Deep convolutional neural networks (CNNs) and U-Net architecture	The study was limited by the analysis of isolated premolars, the absence of real clinical conditions, and difficulties in segmenting thin canals and endodontic pathologies.
Orhan et al., 2020. Reino Unido.	Evaluation of artificial intelligence for detecting periapical pathosis on cone-beam computed tomography scans	The application of AI in endodontics improves the accuracy, speed, and standardization of the diagnosis of periapical lesions in CBCT, assisting in clinical decision-making.	Verify the diagnostic performance of an artificial intelligence system based on the deep convolutional neural network method for detecting periapical pathologies in CBCT images.	Cone Beam Computed Tomography	Deep convolutional neural networks (CNNs) and U-Net architecture	Limitations of the study include the possibility of errors in the segmentation of adjacent lesions, difficulty in distinguishing between lesions and soft tissue due to the low resolution of soft tissue in CBCT, and challenges in analyzing anatomical variations and metallic artifacts.
Santos-Junior, A. O., et al., 2025. Reino Unido.	A novel artificial intelligence-powered tool for automated root canal segmentation in single- rooted teeth on cone-beam computed tomography	Improves accuracy, reduces segmentation time, standardizes diagnoses, and optimizes guided treatment planning.	Develop and validate an artificial intelligence (AI)-based tool for the automatic segmentation of root canals in single-rooted teeth in cone beam computed tomography (CBCT) scans.	Cone Beam Computed Tomography	Deep convolutional neural networks (CNNs) and U-Net architecture	Use of CBCTs from only two devices, exclusion of images with severe artifacts, and the need for validation in more complex cases and in different populations.
Setzer et al., 2020. EUA.	Artificial Intelligence for the Computer-aided Detection of Periapical Lesions in Cone- beam Computed Tomographic Images	Improves diagnostic accuracy, reduces inter-observer variability, enables early detection of periapical lesions, and optimizes analysis time, assisting in clinical decision-making.	Using a Deep Learning (DL) algorithm for automated segmentation of cone beam computed tomography (CBCT) images and detection of periapical lesions.	Cone Beam Computed Tomography	Deep Learning Algorithm with U-Net Architecture	The reliance on clinical segmentation for comparison, the lack of validation with more detailed examinations such as histology, and the need to improve the accuracy of the AI model, especially in the detection of smaller lesions and restorative materials.
Sherwood et al., 2021, Estados Unidos.	A Deep Learning Approach to Segment and Classify C-Shaped Canal Morphologies in Mandibular Second Molars Using Cone- beam Computed Tomography	Accurately identify the anatomy of root canals, making endodontic planning and treatment safer and more effective.	Develop and test deep learning to detect and classify the anatomy of C-shaped canals in lower second molars in CBCT images and compare three deep learning architectures (U-Net, residual U-Net, and Xception U-Net) in terms of Dice index and sensitivity.	Cone Beam Computed Tomography	U-Net, residual U-Net e Xception U-Net	The limitations of this study include the limited sample size and the narrow focus exclusively on the anatomy of C-shaped canals.
Slim et al., 2024. Bélgica.	AI-driven segmentation of the pulp cavity system in mandibular molars on CBCT images using convolutional neural networks	Provide greater precision and speed in endodontic planning, enabling minimally invasive and more efficient treatments.	Develop and validate an artificial intelligence (AI)-based tool for automated segmentation of the pulp cavity system of lower molars in cone beam computed tomography (CBCT) images.	Cone Beam Computed Tomography	Deep convolutional neural networks (CNNs) and U-Net architecture	Use of a relatively small dataset, exclusion of images with intense artifacts, and reliance on CBCTs acquired with specific parameters, which may limit the generalization of results to other devices and clinical conditions.
Wang et al., 2023. China.	Root canal treatment planning by automatic tooth and root canal segmentation in dental CBCT with deep multi-task feature learning	Significantly improve accuracy and reduce endodontic planning time from hours to just minutes in 3D reconstruction for personalized preoperative simulation.	Obtain accurate, automatic segmentation of teeth and root canals from CBCT images, integrating this technology into a clinical workflow to improve surgical planning in endodontic treatments.	Cone Beam Computed Tomography	DentalNet e PulpNet	Reduced number of samples and exclusive focus on single-root teeth, not covering the anatomical variations of root canals in other teeth.
Zhao et al., 2024, China.	An artificial intelligence grading system of apical periodontitis in cone-beam computed tomography data	Improve the diagnosis of apical periodontitis, providing reliable decision support for dentists and optimizing therapeutic planning in endodontics.	Develop a deep learning-based CBCTPAI apical periodontitis (AP) assessment system, evaluating its reliability and accuracy, in order to assist dentists in diagnosing and scoring AP.	Cone Beam Computed Tomography	Development of the self-invented PAINet algorithm	Small sample size, which limits the generalization of the model, and the fact that the system does not have the ability to outperform human experts, being unable to provide correct results for images that are too small or blurred.
